# The Impact of Social Determinants of Health on Facial Synkinesis Severity

**DOI:** 10.1002/lary.70202

**Published:** 2025-10-21

**Authors:** Alexander J. Barna, Amy Wang, Nicole G. DeSisto, Rahul K. Sharma, Scott J. Stephan, Priyesh N. Patel, Shiayin F. Yang

**Affiliations:** ^1^ Drexel University College of Medicine Philadelphia Pennsylvania USA; ^2^ Division of Facial Plastic and Reconstructive Surgery, Department of Otolaryngology‐Head & Neck Surgery Vanderbilt University Medical Center Nashville Tennessee USA; ^3^ Department of Otolaryngology‐Head & Neck Surgery Vanderbilt University Medical Center Nashville Tennessee USA

**Keywords:** facial nerve, facial paralysis, facial synkinesis, health disparities, health equity, non‐flaccid facial paralysis, social determinants of health

## 1 Introduction

Non‐flaccid facial paralysis (NFFP) is a distressing sequela of facial nerve injury that greatly impacts facial function and quality of life [1, 2]. Facial synkinesis is a subtype of NFFP that causes involuntary facial contractions during voluntary facial movements (e.g., eye closure during speech) [3]. Aside from its impact on facial movements, facial synkinesis can also lead to social anxiety, depression, and diminished quality of life [2, 4, 5, 6]. Various treatments exist for the management of NFFP and range from physical therapy to surgical intervention [3, 6, 7]. Facial neuromuscular retraining is a type of physical therapy that is often utilized by patients to retrain facial muscles and improve overall facial function [8]. The main adjunctive treatment for NFFP is chemodenervation with botulinum toxin, which has been shown to improve both facial function and psychological burden [9, 10].

The social determinants of health (SDoH), including geographic, socioeconomic, and structural barriers, influence health outcomes across many health conditions [11], but their impact on facial paralysis has yet to be determined. The aim of this study is to examine how individual and neighborhood SDoH factors affect NFFP outcomes and access to care.

## 2 Methods

This study was approved by the Vanderbilt University Medical Center (VUMC) Institutional Review Board (IRB# 241661) and included patients treated for NFFP at the VUMC Facial Nerve Center from 2017 to 2024. Data collected included facial paralysis etiology, referral patterns, duration of treatment, patient‐reported outcome measures (PROMs) such as the Synkinesis Assessment Questionnaire (SAQ), and physician‐graded scores such as the eFACE. Individual sociodemographic variables included age, sex, and driving time. Neighborhood‐level SDoH factors included the Area Deprivation Index (ADI), a validated measure of neighborhood socioeconomic disadvantage derived from 17 census‐derived indicators (e.g., income, education, housing quality, etc.), and Rural Urban Commuting Area (RUCA) codes, a system from the US Department of Agriculture that categorizes neighborhoods by rurality based on commuting and population density [12, 13]. Descriptive statistics, Mann–Whitney *U* tests, and Wilcoxon signed‐rank tests were used for analysis. All analyzes were conducted using SPSS (IBM Corp, Armonk, NY).

## 3 Results

A total of 213 patients were included. Mean age was 58 ± 14.1 years, 85.4% female and 14.6% male. Most identified as White/Caucasian (81.7%), with smaller proportions identifying as Black African/American (8.5%), Asian (4.2%), and Hispanic/LatinX (1.4%). The mean ADI score was 47.3 (range 3–99) and mean RUCA score was 2.3 (range 1–10). Only 15.1% of patients came from the most deprived neighborhoods (ADI > 75) and 3.9% resided in rural areas or small towns (RUCA ≥ 7).

Paralysis duration averaged 7.5 years (range 0–37); the most common etiologies were Bell's palsy (50.7%) and acoustic neuroma/lateral skull base surgery (22.2%). Referrals came primarily from otology/neurotology (25.3%), self‐referral (22.6%), neurology (11.1%), and family medicine (10.5%), with a trend toward more family medicine referrals among underserved patients (*p* = 0.07). The median time from symptom onset to specialty consultation was 3.5 years (range 0–38), with no difference by deprivation status (3.9 vs. 3.5 years, *p* = 0.45). Over half (58.7%) received physical therapy.

Overall, 49.8% of patients missed at least one chemodenervation appointment; however, the average appointment attendance rate across the cohort was 88.8%. There was no significant difference in the proportion of patients missing at least one appointment between those from underserved areas and those from more advantaged areas (*p* = 0.704). When analyzing the percentage of appointments attended, patients from rural areas demonstrated a significantly higher attendance rate compared with non‐rural patients (95.2% vs. 88.4%, *p* = 0.03).

SAQ and eFACE scores were available for 100 patients. The median SAQ score before chemodenervation was 61.1 and 48.8 after chemodenervation, with a statistically significant median decrease (*z* = −3.078, *p* = 0.002) (Table 1). SAQ scores represent patient quality of life with daily facial movements, with lower scores reflecting better function [14]. Physician‐graded eFACE scores showed median values of 81.5 (resting), 64.0 (dynamic), and 37.5 (synkinesis) domains. Scores for eFACE range from 0 to 100, with higher values indicating better facial function [15]. Patients from more underserved settings demonstrated more severe facial synkinesis. Specifically, patients from socioeconomically deprived neighborhoods had lower eFACE synkinesis scores (median 27.5 vs. 42.5, *p* = 0.03), as did those from rural areas (25.2 vs. 41.2, *p* = 0.02) (Table 1).

**TABLE 1:** Impact of neighborhood socioeconomic deprivation and rurality on eFACE scores.

Variable	ADI ≤ 75	ADI > 75	*p*	RUCA ≤ 6	RUCA ≥ 7	*p*
	Median rank	Median rank		Median rank	Median rank	
Synkinesis Assessment Questionnaire (SAQ)	60.0	64.4	0.972	60.0	64.4	0.598
eFACE dynamic score	64.0	59.0	0.972	64.0	62.5	0.702
eFACE resting score	82.0	75.0	0.986	82.0	70.5	0.108
eFACE synkinesis score	42.5	27.5	0.030	41.3	25.5	0.017

## 4 Discussion

Our findings demonstrate that SDoH factors such as socioeconomic deprivation and rurality are associated with more severe facial synkinesis among patients with NFFP. However, we found that chemodenervation did improve SAQ scores overall for all patients, suggesting quality of life benefit, thus highlighting the importance of improving access to care. We also found that patients from small towns/rural areas had a higher appointment compliance and attended on average 95.2% of their chemodenervation appointments; however, this only accounted for 13 patients, and overall access to care for rural patients appears to be limited.

Past research has found that social disadvantage worsens the severity of health outcomes across multiple conditions in facial plastic surgery, including cleft lip/palate, facial trauma, and more [16]. Although preliminary, the presence of similar disparities with NFFP suggests that structural barriers may be in place that restrict access to specialized care and worsen disease progression. NFFP remains under‐recognized among general healthcare providers, compounding barriers to diagnosis, referral, and treatment [17]. Patients from more deprived areas were less likely to be referred by a specialist, which is likely due to the fact that there are fewer specialists practicing in more deprived neighborhoods [18, 19, 20]. With decreased recognition and referral, patients from underserved communities may suffer greater functional and psychosocial consequences.

Future work is needed to identify the drivers prohibiting access to care for NFFP. Improving equitable outcomes will require strengthening referral pathways, enhancing non‐specialist provider education, and reducing structural barriers that delay access to facial nerve care.

## 5 Conclusion

Patients from socioeconomically deprived and rural areas experienced worse facial synkinesis. However, outcomes improved once patients reached specialty care. Efforts to improve access and treatment equity are critical for optimizing outcomes in this patient population.

## Conflicts of Interest

The authors declare no conflicts of interest.

## Data Availability

The data that support the findings of this study are available on request from the corresponding author. The data are not publicly available due to privacy or ethical restrictions.
